# The Expression and Functional Significance of Runx2 in Hepatocellular Carcinoma: Its Role in Vasculogenic Mimicry and Epithelial–Mesenchymal Transition

**DOI:** 10.3390/ijms18030500

**Published:** 2017-02-27

**Authors:** Zi Cao, Baocun Sun, Xiulan Zhao, Yanhui Zhang, Qiang Gu, Xiaohui Liang, Xueyi Dong, Nan Zhao

**Affiliations:** 1 Department of Pathology, Tianjin Medical University, Tianjin 300070, China; imcaozi@163.com (Z.C.); xiulanzhao@aliyun.com (X.Z.); wyft1022@163.com (Q.G.); liangxiaohui123@126.com (X.L.); dxy7235202@126.com (X.D.); zhaonantj@tmu.edu.cn (N.Z.); 2 Department of Pathology, General Hospital of Tianjin Medical University, Tianjin 300052, China; 3 Department of Pathology, Cancer Hospital of Tianjin Medical University, Tianjin 300060, China; Yanhuizhang015@163.com

**Keywords:** Runx2, Galectin-3, epithelial-mesenchymal transition, vasculogenic mimicry, portal vein invasion, hepatocellular carcinoma

## Abstract

The transcription factor Runx2 has been reported to promote epithelial-mesenchymal transition (EMT) in many tumors. Vasculogenic mimicry (VM) is described as the mimicry of endothelial cells by tumor cells to form microvascular tubes in aggressive tumors. Galectin-3 has been reported to regulate cell invasion, migration, and VM formation; it could be regulated by Runx2. However, the relationship between Runx2, Galectin-3, EMT, and VM has not been studied in hepatocellular carcinoma (HCC). We examined Runx2 expression in 89 human HCC samples and found Runx2 expression was associated with VM. Clinical-pathological data analysis revealed that Runx2 expression was associated with a shorter survival period. Overexpression of Runx2 promoted EMT and enhanced cell migration, invasion, and VM formation in HepG2 cells. Conversely, the downregulation of Runx2 inhibited EMT and reduced cell invasion, migration, and VM formation in SMMC7721. Galectin-3 expression declined following the downregulation of *Runx2* in HepG2 cells, and increased in SMMC7721 cells after *Runx2* knockdown. We consistently demonstrated that the downregulation of *LGALS3* in HepG2-Runx2 cells reduced cell migration; invasion and VM formation; while upregulation of *LGALS3* in SMMC7721-shRunx2 cells enhanced cell migration, invasion, and VM formation. The results indicate that Runx2 could promote EMT and VM formation in HCC and Galectin-3 might have some function in this process.

## 1. Introduction

Primary liver cancer (i.e., hepatocellular carcinoma, HCC) is the fifth most common cancer in men and the seventh in women worldwide, and it represents the third most frequent cause of death from cancer [1]. China is a region in which HCC has a high prevalence. HCC cases and deaths in China accounted for approximately 50% of the total number around the world for just the single year 2012. Surgical resection is the main clinical treatment for HCC. Surgical resection accompanied by liver transplantation improved the disease-free survival in some patients with early stage of HCC, as well as their overall survival [2]. However, the overall prognosis of HCC patients is still unsatisfying. A high potential of intrahepatic recurrence and distant metastasis is responsible for the unsatisfying overall prognosis of HCC patients [3]. During metastasis, the cells lose cell-cell contact as a result of the ablation of E-cadherin and increase their motility to spread into surrounding or distant tissues or organs [4]. Evidence has been found that the epithelial-mesenchymal transition (EMT) plays an important role in the early stage of tumor cell metastasis.

HCC is rich in blood vessels, which could cause aberrant angiogenesis. The growth and metastasis of a tumor depends on its own vascular network. Apart from the vessel tunnels, which are lined with endothelial cells, Maniotis et al. found a new microcirculation mechanism: channels are externally lined with tumor cells, with no endothelial cells in human melanoma [5]. This phenomenon has been termed vasculogenic mimicry (VM). VM is associated with the host blood circulation to provide a blood supply for tumor tissue, which suggests that VM is functional [6]. Early studies in our laboratory showed that VM was found in HCC and that EMT was involved in the formation of VM [7,8,9].

Runx2 is a transcription factor that is a member of the Runx2 family and consists of three Runx genes: *Runx1*/*Cbfa2*/*Pebp2aB*, *Runx2*/*Cbfa1*/*Pebp2aA*, and *Runx3*/*Cbfa3*/*Pebp2aC*. Each Runx gene encodes a domain known as the RUNT domain, which was first characterized in the Runt and Lozenge proteins of *Drosophila* and is essential in many developmental processes. The RUNT domain and CBFβ form the CBF complex that binds to DNA [10,11]. Runx2 was originally cloned from mouse fibroblasts, and its expression has been detected in T-cell lines, NIH3T3 cells, thymus, and the testes. Runx2 is also important in skeletal development [12,13]. Recent studies have found that Runx2 is overexpressed in cancer cells, enhancing their migration and invasion [10,14,15,16,17,18].

Galectin-3 is a β-galactosyl-binding lectin involved in biological functions including cell adhesion, cell migration, cell apoptosis and angiogenesis [19]. Galectin-3 has also been found in many malignant tumors. Researchers discovered that Galectin-3 mediated the expression of VM formation-related genes, such as *VE-cadherin* and *interleukin-8* [20].

Our research has shown that Runx2 expression might promote EMT and induce VM in HCC, and Galectin-3 may function intermediately.

## 2. Results

### 2.1. Runx2 Expression Is Associated with the Presence of VM in HCC

**Figure 1:**
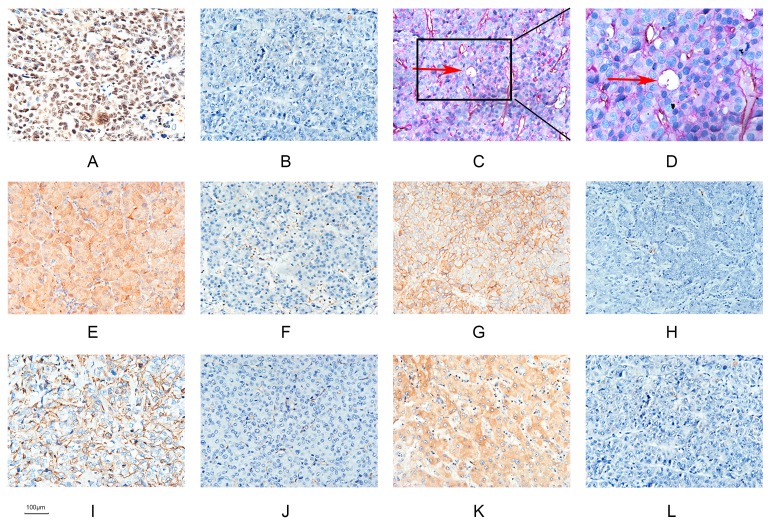
Hepatocellular carcinoma specimens were analyzed by immunohistochemistry. (**A**) Runx2 was predominantly localized in the nuclear of cancer cells (×200; bars 100 µm); (**B**) Negative expression of Runx2 (×200; bars 100 µm); (**C**) CD31/PAS double staining displayed VM channels (Red arrow) in Hepatocellular carcinoma specimens (×200; bars 100 µm); (**D**) VM channels (Red arrow)(×400; bars 100 µm); (**E**) Nuclear and cytoplasmic staining of Galectin-3 (×200; bars 100 µm); (**F**) Negative expression of Galectin-3 (×200; bars 100 µm); (**G**) Positive E-cadherin expression (×200; bars 100 µm); (**H**) Negative E-cadherin expression (×200; bars 100 µm); (**I**) Positive Vimentin expression (×200; bars 100 µm); (**J**) Negative Vimentin expression (×200; bars 100 µm); (**K**) Positive VE-cadherin expression (×200; bars 100 µm); (**L**) Negative VE-cadherin expression (×200; bars 100 µm).

According to the definition of VM, tumor cells mimic endothelial cells to form channels. These unique tubes are lined by tumor cells able to transfer red blood to the surrounding tumor tissue, simultaneously providing a path for tumor cell metastasis [9,12]. VM was found in 53 of 89 HCC samples (59.6%). The relevant clinical pathologic data are shown in Table 1. VM-forming cells were positive for PAS and negative for CD31, indicating that they were HCC cells, not endothelial cells (Figure 1C,D). In the 89 HCC samples, Runx2 expression was found in 55 of 89 (61.8), while VM was detected in 40 out of 55 (72.7%) samples in the Runx2 positive group and 13 out of 34 (38.2%) samples in the Runx2 negative group (Table 2). The difference in the presence of VM in the Runx2-positive and the Runx2-negative group was significant.

### 2.2. Runx2 Expression in HCC Cell Lines, the Induction of Runx2 with Upregulation in HepG2 Cells, and Knockdown in SMMC7721 Cells

**Figure 2:**
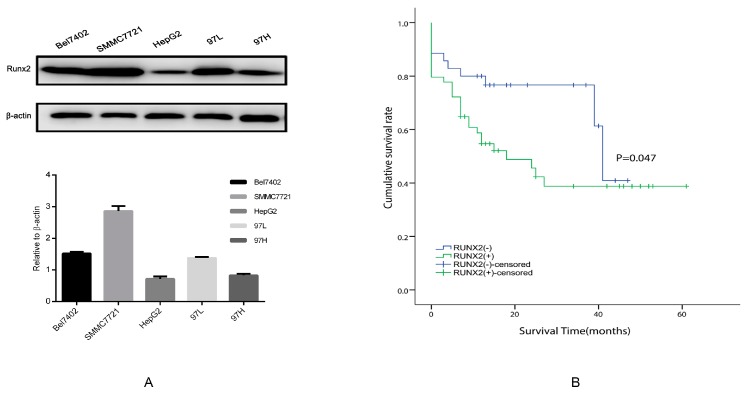
Cell line screening and survival analysis. (**A**) Runx2 protein levels in Bel7402, SMMC7721, HepG2, 97L, 97H cell lines were evaluated by Western blot analysis; (**B**) Overall survival of hepatocellular carcinoma patients with Runx2-positive and Runx2-negative.

**Figure 3:**
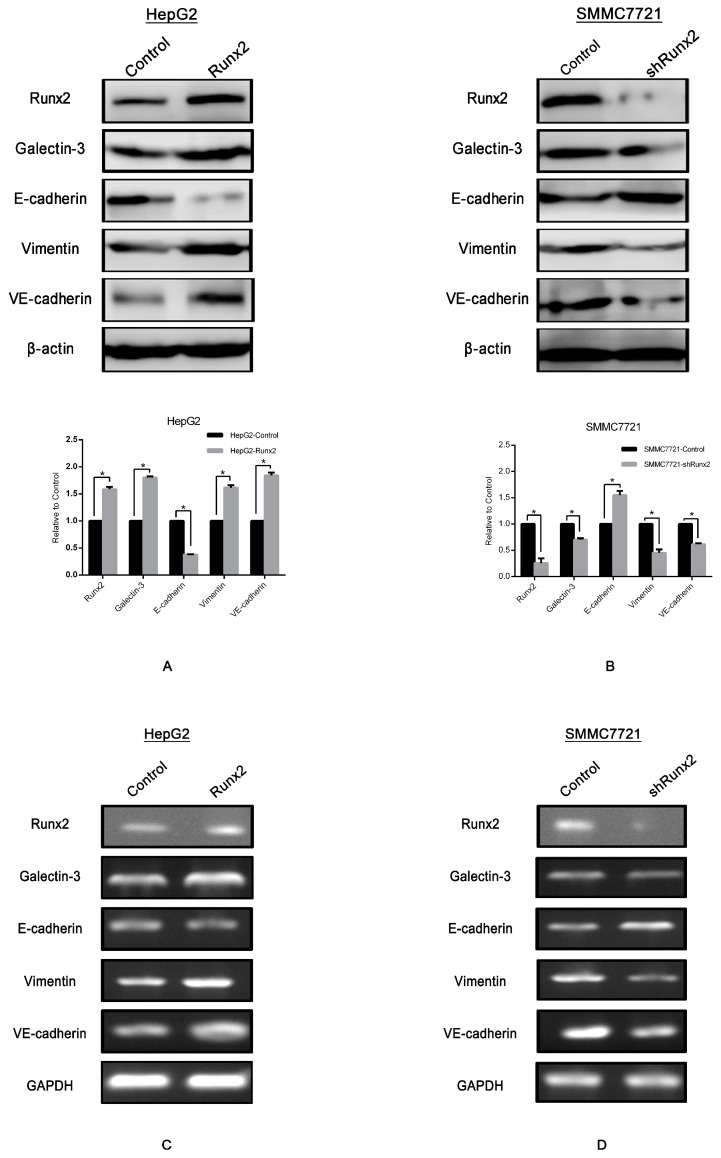
Western blot analysis, β-actin was used as the loading control. Protein levels of Runx2, Galectin-3, E-cadherin, Vimentin, and VE-cadherin in (**A**) HepG2-Runx2 group compared with the HepG2-control group and (**B**) SMMC7721-shRunx2 group compared with the SMMC7721-control group (* *p* < 0.05). RT-PCR analysis, GAPDH was used as the loading control. mRNA levels of Runx2, Galectin-3, E-cadherin, Vimentin, and VE-cadherin in (**C**) HepG2-Runx2 group compared with the HepG2-control group and (**D**) SMMC7721-shRunx2 group compared with the SMMC7721-control group.

To further screen Runx2 expression, we compared the level of the Runx2 protein in various HCC cell lines via Western blotting (Figure 2A). We found that HepG2 had a lowest level of Runx2 expression in contrast to SMMC7721, which had a highest level of expression. HepG2 cells were transfected with the *Runx2* overexpression plasmid. Western blot and RT-PCR revealed an increase in the Runx2 protein and mRNA levels in the HepG2-Runx2 transfectant compared with the control. To further investigate the molecular changes in EMT in the HepG2-Runx2 transfectant, we detected the expression of E-cadherin and Vimentin in the presence of Runx2 over expression compared with control, E-cadherin expression was repressed, however Vimentin expression was raised (Figure 3A,C). In SMMC7721 cells, knockdown by shRNA decreased the Runx2 expression detected with RT-PCR and western blot. The results revealed a high gene knockdown efficiency. When *Runx2* expression was knocked down, E-cadherin expression in SMMC7721-shRunx2 was elevated, and the expression of Vimentin showed the opposite pattern—its expression was suppressed (Figure 3B,D).

### 2.3. Runx2 Upregulation Leads to Increased HCC Cell Invasion and Migration, and VM Formation In Vitro

**Figure 4:**
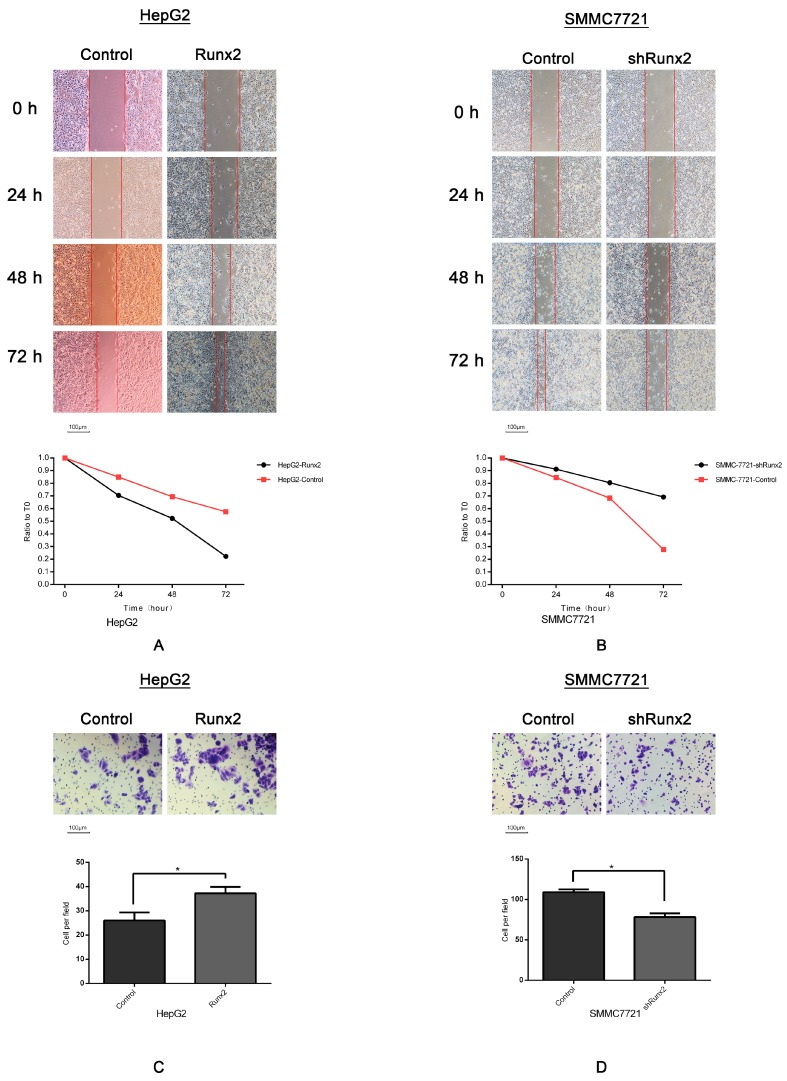
Wound healing assays. (**A**) HepG2-control group and the HepG2-Runx2 group at 0 and 72 h (×40; bars 100 µm); (**B**) SMMC7721-control group and the SMMC7721-shRunx2 group at 0, 24, 48, and 72 h (×40; bars 100 µm). The migration ratio and numbers of migrating cells in the migration assay (×200; bars 100 µm); (**C**) HepG2-control group compared with HepG2-Runx2 group; (**D**) SMMC7721-control group compared with SMMC7721-shRunx2 group. (* *p* < 0.05).

Given the overexpression of the *Runx2* cell model in HepG2 and *Runx2* knockdown cell model in SMMC7721 cells, as well as the detection of downregulation of E-cadherin, and upregulation of Vimentin in HepG2-Runx2 transfectants, upregulation of E-cadherin and downregulation of Vimentin in SMMC7721-shRunx2 transfectants, we investigated the effect of Runx2 expression on cell invasion, migration, and VM formation. Cell migration and invasion were prone to VM formation, as indicated by studies in our laboratory [9,12], and tumor cells that underwent EMT to form VM channels gained an increased ability to invade and metastasize. We studied the invasion and migration ability of HepG2 and SMMC7721 cells after *Runx2* upregulation and knockdown. Quantitative analysis following a wound healing assay suggested a significant difference in the speed of wound healing between the transfected groups and the control group (Figure 4A,B). Following a Matrigel invasion assay, an increase in cell invasion was observed in the Runx2-transfected HepG2 cell line compared with the control, and a decrease in cell invasion in the Runx2-shRNA-transfected SMMC7721 cells compared with the control (Figure 4C,D) (*p* < 0.05).

**Figure 5:**
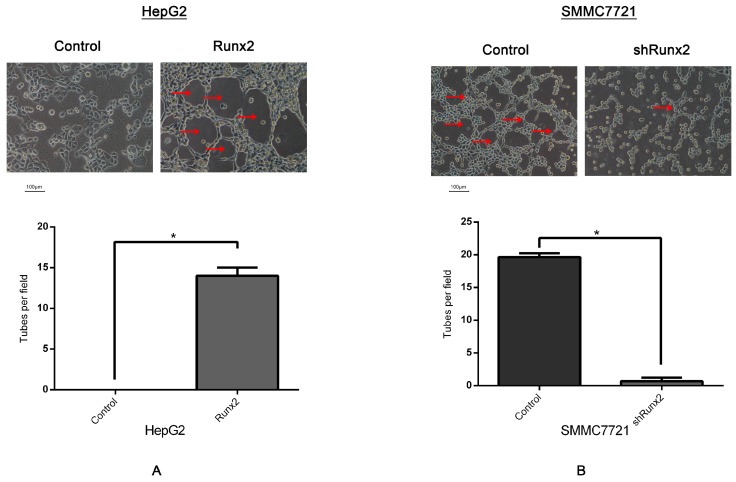
Three-dimensional culture on Matrigel matrix (×200; bars 100 µm), VM-like tubes are pointed by Red arrows (**A**) VM-like tube formation in HepG2-Runx2 compared with HepG2-control cells; (**B**) decreased number of VM formation of SMMC7721-shRunx2 compared with SMMC7721-control cells. (* *p* < 0.05).

**Figure 6:**
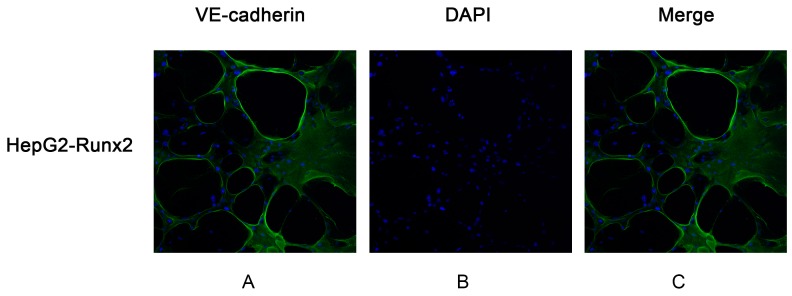

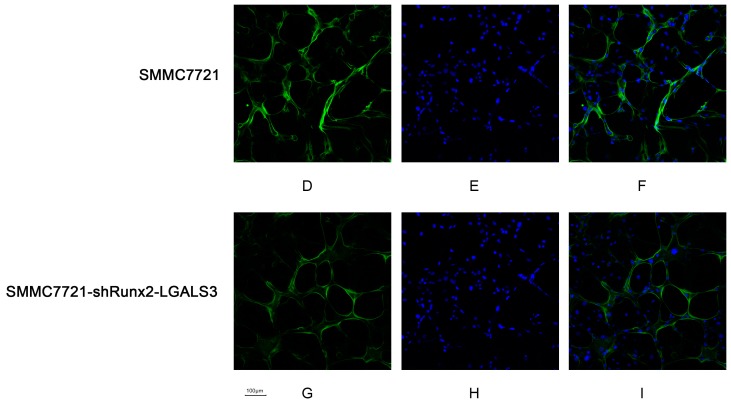
The VM-like tubes formed by HepG2-Runx2 cells, SMMC7721 cells and SMMC7721-shRunx2-LGALS3 cells were assessed by VE-cadherin immunofluorescence and confocal microscopy (×200). (**A**,**D**,**G**) VE-cadherin staining of the VM channel was concentrated in the wall of the tubes; (**B**,**E**,**H**) The cell nuclei were stained by DAPI; (**C**,**F**,**I**) The merged images showed that the expression of VE-cadherin in VM networks.

The Matrigel 3D culture was used to investigate vasculogenic mimicry in vitro to determine whether Runx2 mediated the morphological alteration of the HCC cells. Our results demonstrated that when up- or downregulated by Runx2, HepG2 and SMMC7721 cells formed a typical pipe-like structure on the surface of the Matrigel medium (Figure 5). No significant differences were found for the HepG2 cells regarding the efficiency of network formation, but SMMC7721 cells formed a network which could be observed in the controls. A lack of tube formation was evident in the HepG2 control (Figure 5A), providing support for a possible role of Runx2 in promoting VM formation. Compared with the control, a significant decrease of VM formation was noted in SMMC7721 cells following *Runx2* knockdown (Figure 5B). Interestingly, those tube-like structures overexpressed the endothelial-specific marker VE-cadherin, especially on the wall of these tubes in vitro (Figure 6C,F). These results indicated that Runx2 expression could be related to HCC cell invasion and migration, and overexpression of Runx2 might lead to an increase in the invasiveness and migration of HCC cells, and Runx2 could play an important role in VM formation in vitro.

### 2.4. Runx2 Promotes Galectin-3 Expression

Galectins are a family of well-conserved carbohydrate-binding proteins. Galectin-3 is a chimera-type 31 kDa galactose-binding protein consisting of a short NH_2_-terminal domain and a carboxy-terminal domain. Galectin-3 regulated the expression of VE-cadherin, and has been reported to play an essential role during the formation of VM in tumor cells [20,21]. VE-cadherin is expressed in highly aggressive tumor cells and is an important marker of VM [22]. Further studies in our laboratory indicated the upregulation of Galectin-3 and VE-cadherin expression in HepG2-Runx2 cells compared to the control (Figure 3A,C), and its downregulation in SMMC7721-shRunx2 compared to the control (Figure 3B,D). The results indicated that both VE-cadherin and Galectin-3 expression were increased in the 3D culture system based on the *Runx2* transfection of HepG2 cells compared with the control group (Figure 5A). We used a shRNA-based technique to silence *Runx2* expression in SMMC7721 cells, and the results showed the downregulation of Runx2 as well as a significant decrease in VM formation in the 3D cultures compared with the control (Figure 5B), with a concomitant decrease in Galectin-3 and VE-cadherin. These results suggested that Runx2 expression might promote the expression of Galectin-3 in vitro.

### 2.5. LGALS3 Knockdown in HepG2-Runx2 Cells, and Its Upregulation in SMMC7721-shRunx2 Cells

**Figure 7:**
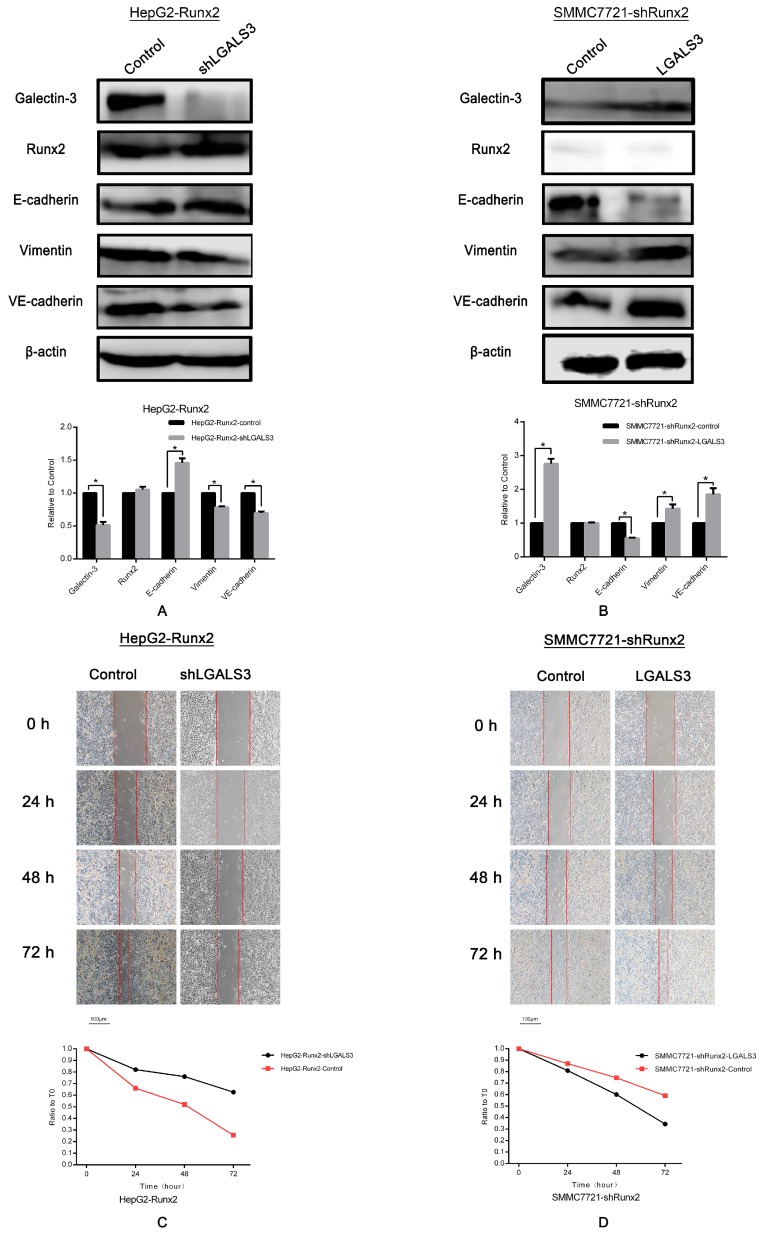

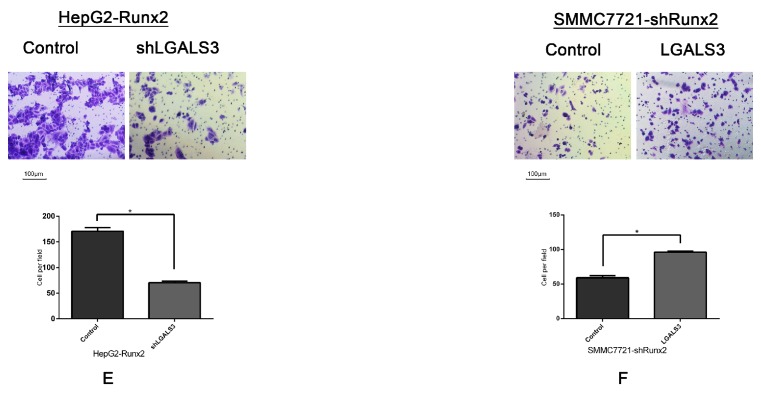
Western blot analysis, β-actin was used as the loading control. (**A**) Protein levels of Galectin-3, Runx2, E-cadherin, Vimentin, and VE-cadherin after *LGALS3* knockdown in HepG2-Runx2 cells; (**B**) Transfection of *LGALS3* plasmid in SMMC7721-shRunx2 cells; protein levels of Galectin-3, Runx2, E-cadherin, Vimentin, and VE-cadherin. Wound healing assays. (**C**) HepG2-Runx2-control group and the HepG2-Runx2-shLGALS3 group at 0 and 72 h (×40; bars 100 µm); (**D**) SMMC7721-shRunx2-control group and the SMMC7721-shRunx2-LGALS3 group at 0, 24, 48, and 72 h (×40; bars 100 µm). The migration ratio and numbers of migrating cells in the migration assay (×200; bars 100 µm); (**E**) HepG2-Runx2-control group compared with HepG2-Runx2-shLGALS3 group; (**F**) SMMC7721-shRunx2-control group compared with SMMC7721-shRunx2-LGALS3 group (* *p* < 0.05).

Given the upregulated Galectin-3 level in *Runx2* upregulated HepG2 cells, and downregulation in *Runx2* knockdown SMMC7721 cells, we then performed a Galectin-3 gene *LGALS3* knockdown in HepG2-Runx2, and an upregulation in SMMC7721-shRunx cells to determine whether Galectin-3 is responsible for the effect of Runx2 expression on EMT and VM in HCC cells. Western blot showed that the Galectin-3 expression level in HepG2-Runx2 cells decreased following *LGALS3* knockdown compared with the control (Figure 7A), and increased as a result of the *LGALS3* transfection of SMMC7721-shRunx2 cells compared with the control (Figure 7B). However, no significant change in Runx2 expression was observed after *LGALS3* transfection or knockdown compared with the control (Figure 7A,B), indicating that Galectin-3 expression might not affect Runx2 expression in HCC cells.

We then investigated the expression of E-cadherin and Vimentin in *LGALS3* down- or upregulated cell lines to examine whether Galectin-3 affected the EMT process. We observed nearly opposite results compared with the results of the *Runx2* up- or downregulation experiments. E-cadherin expression increased while Vimentin expression was downregulated in HepG2-Runx2-shLGALS3 cells compared with control (Figure 7A), and E-cadherin expression in SMMC7721-shRunx2-LGALS3 cells decreased, but the expression of Vimentin increased (Figure 7B). The results indicated that EMT was inhibited in HepG2-Runx2 cells after *LGALS3* knockdown, and EMT was stimulated in Galectin-3 upregulated SMMC7721-shRunx2 cells, which suggested that Runx2 might regulate EMT in HCC cells via Galectin-3, which functioned intermediately in HCC cells.

### 2.6. Galectin-3 Promoted HCC Cell Invasion, Migration, and VM Formation In Vitro

Based on the effect of Galectin-3 on regulating EMT, we then studied the invasion and migration, as well as VM formation ability of HepG2-Runx2-shLGALS3 and SMMC7721-shRunx2-LGALS3 cells to determine whether Galectin-3 could affect those cellular functions. Wound healing assays showed that *LGALS3* knockdown slowed the wound healing in HepG2-Runx2 cells compared with the control (Figure 7C), and the introduction of *LGALS3* increased wound healing rapidity in SMMC77210-shRunx2 cells (Figure 7D). After a Matrigel invasion assay, we observed a decrease in HepG2-Runx2 cells after *LGALS3* knockdown (Figure 7E), and an increase in SMMC7721-shRunx2 cells following *LGALS3* upregulation compared with the control (Figure 7F).

**Figure 8:**
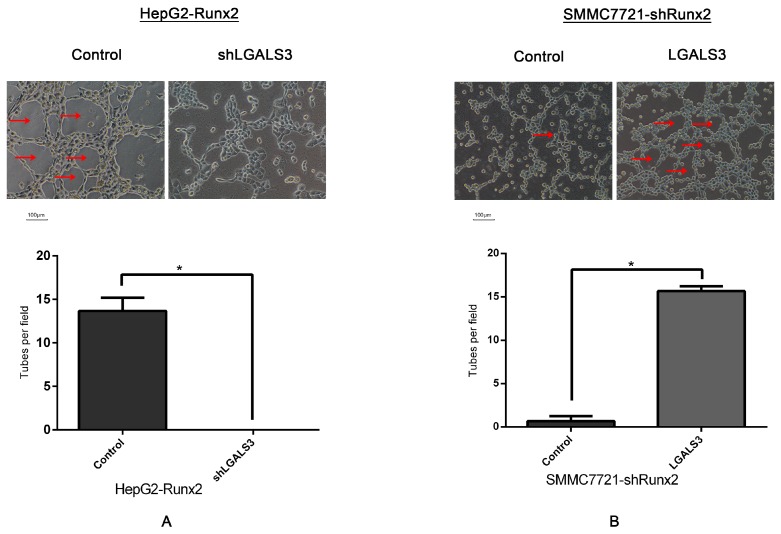
Three-dimensional culture on Matrigel matrix (×200; bars 100 µm), VM-like tubes are pointed by Red arrows. (**A**) VM-like tube formations disappear in HepG2-Runx2-shLGALS3 compared with HepG2-Runx2-control cells; (**B**) increased number of VM formation of SMMC7721-shRunx2-LGALS3 compared with SMMC7721-shRunx2-control cells (* *p* < 0.05).

Furthermore, we found VE-cadherin expression was downregulated after *LGALS3* knockdown in HepG2-Runx2 cells (Figure 7A), and upregulated following *LGALS3* upregulation in SMMC7721-shRunx2 cells (Figure 7B). Three-dimensional culture experiments were performed to investigate VM formation to determine whether Galectin-3 could affect the formation of VM-like structures. Our results demonstrated that HepG2-Runx2-shLGALS3 cells lost the ability to form tube-like structures compared with the control (Figure 8A), while the number of such tube-like structures was higher in SMMC7721-shRunx2 cells compared with the control after *LGALS3* upregulation (Figure 8B); those tube-like structures also overexpressed VE-cadherin, especially on the wall of these tubes in vitro (Figure 6I). These results indicated that Galectin-3 might play an important role in cell invasion and migration, as well as in VM formation in HCC cells, and that Runx2 functioned via Galectin-3.

### 2.7. The Relationship between Runx2, VM-Related Markers, Galectin-3, and Clinical Data

Immunohistochemical analyses were used to assess the expression of E-cadherin, Vimentin, VE-cadherin, and Galectin-3 in the 89 HCC samples. The results showed that Runx2, Galectin-3, E-cadherin, Vimentin, and VE-cadherin expression was located in the nucleus, cytoplasm, or cell membrane of HCC cells (Figure 1A,E,G,I,K). The expression of these proteins in cells with or without Runx2 was compared with a χ^2^ test. The positive rate of Vimentin, VE-cadherin, and Galectin-3 expression in the presence of Runx2 was higher than in cancer cells that did not express Runx2, while the positive rate of E-cadherin in Runx2-positive cells was lower than in the Runx2-negative group. The differences were statistically significant (Table 2) (*p* < 0.05). The Runx2 positive rate was 13 out of 30 (43.3%) in group I, 21 out of 30 (70%) in group II, and 21 out of 29 (72.4%) in group III (Table 1). A Kaplan-Meier survival analysis revealed that patient with Runx2 expression had a shorter survival period than those without expression (Figure 2B).

## 3. Discussion

The Runx (Runt-related transcription factor) genes comprise three closely related transcription factors: *Runx1/Cbfa2/Pebp2aB*, *Runx2/Cbfa1/Pebp2aA*, and *Runx3/Cbfa3/Pebp2aC*. The three genes are characterized by the RUNT box, which is a highly conserved 128 amino acid DNA binding/protein-protein interaction domain homologous with the Drosophila pair-rule gene RUNT. Each Runx protein has a unique function: Runx1 is essential for hematopoiesis, Runx3 is important for nerve cell development, and Runx2 is a key regulator of bone development. A *Runx2*-deficient mice model showed a complete lack of bone formation [10,14,17]. Recent studies have shown that Runx2 was associated with tumor cell invasion and metastasis. Boregowda and colleagues demonstrated that a reduction in Runx2 activity led to decreased growth, migration, and invasion in melanoma cells [14]. Baniwal et al. found that Runx2 promoted invasion and metastasis to bone in prostate cancer cells [23]. Also, Runx2 was shown to be a key transcription factor that promoted the metastasis of colorectal carcinoma cells [24]. Runx2 could be a potential target for tumor cells prone to spread to distant organs via invasion and metastasis.

Hepatocellular carcinoma (HCC) is a malignant tumor with a high incidence of intrahepatic and extrahepatic recurrence after resection, which contributes to the poor prognosis of HCC patients. Evidence suggests that portal vein invasion (PVI) occurs in the early stage of HCC and is associated with metastasis [2,25,26]. In HCC samples, the Runx2 positive rate in group II was higher than in group I, and highest in group III, which might indicate that Runx2 is associated with HCC cell metastasis. Jue and colleagues reported that vasculogenic mimicry (VM) contributes to HCC PVI [25]. The existence of VM in melanoma was first reported by Maniotis and colleagues; since then VM has been found in many types of tumors including breast carcinoma, hepatocellular carcinoma, colon carcinoma, lung cancer, etc. Previous studies in our laboratory revealed that VM was associated with metastasis in HCC and the occurrence of VM is associated with a poor prognosis [27]. VM channels can provide blood to tumors and expose tumor cells directly to the bloodstream, which facilitates tumor cell metastasis to surrounding or distant tissues. The lining of VM channels in HCC tumor cells is derived from epithelial cells. The transition into endothelial cells is similar to an epithelial-mesenchymal cell transition. EMT is a process by which epithelial cells lose their epithelial characteristics and gain mesenchymal characteristics, acquiring the ability to invade and metastasize [28,29]. During EMT, the expression of epithelial markers such as E-cadherin are downregulated and the expression of mesenchymal markers such as Vimentin is upregulated [22,30]. Tumor cells that have undergone EMT have an increased ability to metastasize. Our previous studies indicated that the EMT plays important role in VM formation in HCC [9,12]. Emerging evidence has shown that Runx2 could promote EMT [21,31].

In our study using human HCC tissue samples, we found that Runx2 nuclear expression was associated with the presence of VM, E-cadherin, and Vimentin. Patients with Runx2 expression had a shorter survival period. We first transfected HepG2 cells with a *Runx2* overexpression plasmid and a control plasmid, and SMMC7721 cells with a *Runx2* shRNA plasmid and a control plasmid. Increased and decreased Runx2 expression were observed in HepG2-Runx2 and SMMC7721-shRunx2 cells, respectively. In HepG2-Runx2 cells, E-cadherin expression was decreased, and Vimentin expression was increased compared to the control, thus inducing EMT. In SMMC7721-shRunx2 cells, E-cadherin expression was increased, and Vimentin was decreased compared to the control, thus EMT was inhibited. A biological function test demonstrated that an increase in Runx2 expression could promote the invasion and migration of HepG2 cells compared to the control. In SMMC7721 cells, a *Runx2* knockdown led to less invasion and migration compared to the control. The in vitro data showed that Runx2 plays an important role in tumor cell invasion and migration.

Vascular endothelial (VE)-cadherin is an endothelial specific adhesion molecule located at junctions between endothelial cells. In addition to its adhesive functions, VE-cadherin is essential during embryonic angiogenesis [32]. Studies showed that VE-cadherin is usually present during VM formation in tumor cells and is a significant marker for VM, exclusively expressed in highly aggressive tumor cells [33]. In addition to its function as an adhesive transmembrane protein, it can lead to the induction of a vascular signaling cascade. In the human HCC samples, VE-cadherin expression was associated with Runx2 expression. We further examined the expression of VE-cadherin and found that its expression in HepG2-Runx2 cells was upregulated compared to the control, and downregulated in SMMC7721-shRunx2 cells. Additionally, three-dimensional culture showed that, after transfection, HepG2-Runx2 cells contained VM-like structures on the surface of the Matrigel surface, as compared to the control. The number of VM tube-like structures was lower in SMMC7721-shRunx2 cells compared to the control. The in vitro data showed that Runx2 might play an important role in tumor cell invasion and migration and be closely associated with VE-cadherin expression and tumor cell plasticity to VM pattern.

Galectin-3 is in the β-galactoside-binding lectin family, which is characterized by a conserved sequence defined by a structural similarity in the carbohydrate-recognition domain. Galectin-3 has three motifs: a short NH_2_ terminal domain that contains a serine phosphorylation site, a repetitive proline-rich collagen-α-like sequence that can be cleaved by matrix metalloproteases, and a globular COOH-terminal domain containing a carbohydrate-binding motif and an NWGR anti-death motif [19]. The expression of Galectin-3 in HCC is associated with poor prognosis [34]. Galectin-3 is involved in many biological activities including tumor metastasis and tumor angiogenesis. The accumulating of Galectin-3 in the tumor cell cytoplasm stimulates the tumor cell to become more invasive and provoke distant metastasis. Studies have indicated that Galectin-3 mediates gene expression related to VM formation, such as VE-cadherin [20,35]. In addition, Wang and colleagues found that the overexpression of Galectin-3 upregulated the expression of the Wnt protein, activated β-catenin, and induced EMT [36]. Studies of the relationship between Runx2 and Galectin-3 demonstrated that Runx2 mediated the expression of Galectin-3 in skeletal tissue, human glioma cells, and human pituitary tumor [2,37,38]. We noticed that, in HCC samples, Runx2 expression is related to Galectin-3 expression (χ^2^ = 5.92, *p* = 0.018); furthermore, we examined the Galectin-3 expression level in HepG2 and SMMC7721 cells Runx2 transfected models. The Galectin-3 expression level was upregulated in HepG2 cells after *Runx2* was upregulated, and downregulated in SMMC7721 cells following *Runx2* knockdown. Because of these findings, we hypothesized that Runx2 may mediate Galectin-3, affecting EMT and VE-cadherin, and finally leading to increased tumor cell invasion, migration, and VM formation in HCC. So we transfected Runx2-upregulated HepG2 cells with a *LGALS3* shRNA plasmid containing the Galectin-3 gene and a control plasmid, and *Runx2* knockdown SMMC7721 cells with a *LGALS3* overexpression plasmid and a control plasmid. Decreased and increased Galectin-3 expression were detected in HepG2-Runx2-shLGALS3 and SMMC7721-shRunx2-LGALS3 cells. We then examined the expression of E-cadherin, Vimentin, and VE-cadherin in each cell type. In HepG2-Runx2-shLGALS3 cells, E-cadherin expression was increased and Vimentin expression was decreased compared to the control. In SMMC7721-shRunx2-LGALS3 cells, E-cadherin expression was decreased and Vimentin expression was increased. Galectin-3 downregulation in Runx2 upregulated HepG2 cells inhibited EMT, and Galectin-3 upregulation in Runx2 downregulated SMMC7721 cells stimulated EMT.

An investigation of the biological functions indicated that the HepG2-Runx2 cells showed a decreased invasion and migration ability after Galectin-3 downregulation. In SMMC7721-shRunx2 cells, we found greater cell invasion and migration following the knockdown of Galectin-3.

No significant changes in Runx2 expression were observed after transfection compared to the control. The results indicated that Galectin-3 could be a downstream regulator of Runx2. We further investigated the expression of VE-cadherin, which was reported to be regulated by Galectin-3. Its expression in HepG2-Runx2-shLGALS3 cells was downregulated compared to the control, and upregulated in SMMC7721-shRunx2-LGALS3 cells compared to the control. The ability to form VM-like structures was lost after Galectin-3 was downregulated in HepG2-Run2 cells compared to the control. In SMMC7721 cells, the number of VM-like channels was higher compared with the control. These data showed that Galectin-3 could be a key point in VE-cadherin expression and was crucial for the formation of VM.

## 4. Materials and Methods

### 4.1. Patient Samples

HCC specimens were obtained from 89 patients who received surgical excision in Tianjin General Hospital and Tianjin Medical University Cancer Hospital from 2005 to 2015. All cases were categorized into three groups. The first 30 patients were diagnosed as small hepatocellular carcinoma (≤3 cm) with no lymphovascular invasion and intrahepatic metastasis, the second 30 cases were found to have portal vein invasion during surgery but with no extrahepatic metastases, and the last 29 patients were reported to have extrahepatic metastasis after surgical treatment. The diagnoses were verified by two individual pathologists. The clinical data were collected following a previously employed protocol [12], including gender, age, tumor size, histological differentiation, metastasis, stage, and survival duration. All specimens came from patients who received no radiotherapy or chemotherapy. All subjects gave their informed consent for inclusion before they participated in the study. The study was conducted in accordance with the Declaration of Helsinki, and the protocol was approved by the Ethics Committee of Tianjin Medical University with study number TMUHMEC2015006.

### 4.2. Antibodies and Reagents

Anti-Vimentin antibody (ab92547), anti-VE-cadherin antibody (ab33168), and anti-Runx2 antibody (ab92547) were purchased from Abcam (Shanghai, China). Galectin-3 antibody (sc-20157) was purchased from Santa Cruz Biotechnology (Santa Cruz, CA, USA). Transwell cell inserts were purchased from Falcon Co. (Durham, NC, USA). Matrigel was purchased from BD Biosciences (San Jose, CA, USA).

### 4.3. Immunohistochemical (IHC) and Histochemical Double Staining Method

The specimen sections were pretreated with xylene, absolute ethyl alcohol, and methanol. Hydrogen peroxide (3%) was used to block internal peroxidase. Microwave heating was used for antigen retrieval. The staining systems were PicTure PV6000 (Zhongshan Chemical Co., Beijing, China) and Elivision Plus (Zhongshan Chemical Co.). Phosphate-buffered saline (PBS) was used instead of a primary antibody as a negative control. After IHC staining for endomucin, periodic acid-Schiff (PAS) staining was performed. IHC staining quantification criteria: Positive rate: select 10 views (×400) randomly and calculate positive rate per 100 cells, 0 point (below 10%), 1 points (10%–25%), 2 points (25%–50%), 3 points (above 50%). Immunostaining intensity: 0 point (negative), 1 points (faint yellow), 2 points (deep yellow), 3 points (brown yellow). Staining Index was product of positive rate and immunostaining intensity. Specimens with a staining index above or equal to 3 points were marked as positive; those lower than 3 points were marked as negative.

### 4.4. Cell Culture

HepG2 and SMMC7721 cell lines were obtained from the American Type Culture Collection and the Fudan University-affiliated Zhongshan Hospital (Shanghai, China). The cells were cultured in Dulbecco’s modified Eagle’s medium supplemented with 10% fetal bovine serum.

### 4.5. Runx2 Plasmids

A *Runx2* overexpression plasmid (Catalog No.: EX-H5214-Lv201), an overexpression control plasmid (Catalog No.: EX-NEG-Lv201), shRNA against the *Runx2* plasmid (Catalog No.: HSH021333-LVRU6GP), and a shRNA control plasmid (Catalog No.: CSHCTR001-LVRU6GP) were purchased from Genecopia Co. (Rockville, MD, USA). The stable selection marker was puromycin.

### 4.6. LGALS3 Plasmids

An *LGALS3* overexpression plasmid (Catalog No.: EX-O0067-Lv155), anover expression control plasmid (Catalog No.: EX-NEG-Lv155), shRNA against the *LGALS3* plasmid (Catalog No.: HSH010590-LVRH1MH), and a shRNA control plasmid (Catalog No.: CSHCTR001-LVRU6MH) were purchased from Genecopia Co. The stable selection marker was hygromycin.

### 4.7. Transfection

The cell lines were transfected by following the protocol for the Lenti-Pac™ HIV expression kit (Genecopia Co.) HepG2 cells were first transfected with the *Runx2* overexpression plasmid to upregulate Runx2, and then transfected with the *LGALS3* shRNA plasmid to downregulate Galectin-3. SMMC7721 cells were first transfected with the *Runx2* shRNA plasmid to downregulate Runx2, and transfected with the *LGALS3* overexpression plasmid to upregulate Galectin-3. The negative control plasmids were transfected at the same time. The transfected cells were treated with puromycin (Sigma, St. Louis, MO, USA) and hygromycin B (Roche, Basel, Switzerland).

### 4.8. Semi-Quantitative RT-PCR

The total cellular RNA was extracted using TRNzol-A^+^ reagent (Tiangen Biotech, Beijing, China) according to the manufacturer’s instructions. Before semi-quantitative RT-PCR, 2 µg of total RNA were reversed transcribed to cDNA in a 25 µL reaction system using the QuantScript RT Kit (Tiangen Biotech). The 2X Taq PCR MasterMix (Tiangen Biotech) was used for the PCR reaction system. RT-PCR was performed according to the recommended profile: preincubation at 95 °C for 3 min, denaturation at 95 °C for 30 s, annealing for 30 s, followed by elongation at 75 °C for 1 min. A gradient PCR was used with an annealing temperature for Human *Runx2* (40 cycles) of 58.6 °C and for Human *LGALS3* (30 cycles) of 55.9 °C. The annealing temperature for *E-cadherin* (30 cycles) was 56 °C, 58.7 °C for *Vimentin* (30 cycles), 57.5 °C for *GAPDH* (30 cycles), and 56 °C for *VE-cadherin* (30 cycles). Each amplified product (5 µL) was subjected to electrophoresis in a 1% agarose gel mixed with ethidium bromide (Bio-Rad, Hercules, CA, USA). The expression of *GAPDH* was used for standardize the amount of RNA in each sample. Primer information is supplied in Table 3.

### 4.9. Western Blot Analysis

HepG2 and SMMC7721 cells were first washed with PBS, then lysed with 10% SDS. After SDS-PAGE electrophoresis, the cell lysates were transferred onto PVDF membranes (Millipore, Billerica, USA). These membranes were blocked in 5% skim milk and incubated overnight at 4 °C with the primary antibodies (E-cadherin, 1:200; Vimentin, 1:1000; VE-cadherin, 1:500; β-actin, 1:2000; Galectin-3, 1:200; Runx2, 1:500).The secondary antibodies were added and incubated at 37 °C for 2 h, and an enhanced chemiluminescence method was then performed to measure protein expression. Equal sample loading was confirmed by probing the membranes with a β-actin antibody; the bands were assessed by a Blot Scanner (LI-COR, Hong Kong, China) and analyzed using ImageJ (1.49v, National Institutes of Health, Bethesda, MD, USA).

### 4.10. Transwell Assay

The Transwell upper chambers were coated with Matrigel in advance before being placed in 24-well plates. The lower chambers were filled with 10% complete Dulbecco’s modified eagle medium (DMEM). HepG2 and SMMC7721 cells were plated in the upper chambers in a DMEM medium containing no FBS. The cells then invaded the Matrigel matrix and adhered to the bottom surface of the membrane. The cells on the top surface of membrane were cleared out, then the cells on the bottom surface of the membrane were fixed with cold methanol and stained with 0.5% crystal violet. We selected 10 views randomly and the number of invading cells was counted under an inverted light microscope.

### 4.11. Migration Assay

A wound-healing assay was used to investigate the migration ability of HepG2, SMMC7721, and Bel740 cells. The cells were first plated into 24-well culture plates in a monolayer, and then a straight scratch in the center of each well was made using a micropipette. Cell motility was evaluated by measuring the movement of cells into the scratch. The scratch closure was recorded at 0, 24, 48, and 72 h and the scratch length was compared to the 0 h measurement.

### 4.12. Three-Dimensional Culture

A three-dimensional culture was used to evaluate the capacity for VM formation in vitro. The cells were cultured in 96-well plates coated with Matrigel matrix (20 µL). HepG2 and SMMC7721 cells were seeded into the gel in complete DMEM medium and incubated for 24 h; the cell numbers of treatment and control group bore no difference. We selected 10 views randomly, the formation of tube-like structures was captured, and the number was counted under an inverted light microscope.

### 4.13. VE-Cadherin Immunofluorescence and Confocal Microscopy

Cells were seeded on coverslips until they formed VM-like structures. Then the cells were fixed with cold methanol for 20 min and blocked with 5% FBS. The coverslips were incubated with VE-cadherin primary antibody (1:40) overnight at 4 °C and incubated with secondary antibody at 37 °C for 2 h. The nuclei were stained with DAPI (Zhongshan Chemical Co., Beijing, China). The cells were viewed under a confocal microscope (Nikon A1, Tokyo, Japan).

### 4.14. Statistical Analysis

The data were analyzed in SPSS 17.0 (SPSS Inc., Chicago, IL, USA). *p* < 0.05 was considered statistically significant. A chi-square test was used to analyze the clinical data. A Kaplan-Meier survival analysis was performed, and a log-rank test was used to examine the differences in the survival curves.

## 5. Conclusions

In conclusion, we first showed that Runx2 could promote EMT and induce VM in HCC, in which Galectin-3 might have some function in this process. Via the regulation of Galectin-3 expression, Runx2 was associated with EMT and VM. Further studies are required to elucidate the mechanism behind the relationship between Runx2 and Galectin-3.

## Figures and Tables

**Table 1:** Correlation between Runx2 expression and clinicopathologic parameters, and grouping.

Factors	Runx2		χ^2^	*p*
	Positive	Negative		
Age (years)				
≤45	6	6	0.818	0.366
>45	49	28		
Gender				
Male	46	29	0.044	0.835
Female	9	5		
Hepatitis B				
Positive	31	18	0.099	0.752
Negative	24	16		
Cirrhosis				
Positive	27	18	0.125	0.724
Negative	28	16		
Tumor size (cm)				
≤5	21	18	3.382	0.066
>5	34	16		
Histological differentiation				
I/II	21	22	6.994	0.008 *
III/IV	34	12		
Metastasis				
Positive	42	17	8.106	0.004 *
Negative	13	17		
Group				
I	13	17	6.572	0.037 *
II	21	9		
III	21	8		

* Significantly different.

**Table 2:** Correlation between Runx2 expression with E-cadherin, Vimentin, VE-cadherin, VM, and Galectin-3.

Variant	Runx2		χ^2^	*p*
	Positive	Negative		
E-cadherin				
Positive	18	20	5.848	0.027 *
Negative	37	14		
Vimentin				
Positive	29	6	10.837	0.002 *
Negative	26	28		
VE-cadherin				
Positive	37	9	14.008	0.001 *
Negative	18	25		
VM				
Positive	40	13	10.337	0.002 *
Negative	15	21		
Galectin3				
Positive	34	12	5.92	0.018 *
Negative	21	22		

* Significantly different.

**Table 3:** RT-PCR primer information.

Gene	Forward Primer	Reverse Primer
*Runx2*	5′-CTCAGTGATTTAGGGCGCAT-3′	5′-CTGGCTCTTCTTACTGAGAG-3′
*LGALS3*	5′-GGCCACTGATTGTGCCTTAT-3′	5′-TGCAACCTTGAAGTGGTCAG-3′
*E-cadherin*	5′-AAACAGGATGGCTGAAGGTG-3′	5′-TCAGGATCTTGGCTGAGGAT-3′
*Vimentin*	5′-TGGCACGTCTTGACCTTGAA-3′	5′-GGTCATCGTGATGCTGAGAA-3′
*VE-cadherin*	5′-CTTCTCTGCCTCACCTGGTC-3′	5′-GCCACTTCTCCAAGGTGTGT-3′
*GAPDH*	5′-CCTGGCCAAGGTCATCCATGAC-3′	5′-TGTCATACCAGGAAATGAGCTTG-3′

## Abbreviations

HCC	Hepatocellular Carcinoma
EMT	Epithelial-Mesenchymal Transition
VM	Vasculogenic Mimicry
PAS	Periodic Acid-Schiff
PVI	Portal Vein Invasion
