# Correction: Cao, Z., et al. The Expression and Functional Significance of Runx2 in Hepatocellular Carcinoma: Its Role in Vasculogenic Mimicry and Epithelial—Mesenchymal Transition. *Int. J. Mol. Sci.* 2017, *18*, 500

**DOI:** 10.3390/ijms22010077

**Published:** 2020-12-23

**Authors:** Zi Cao, Baocun Sun, Xiulan Zhao, Yanhui Zhang, Qiang Gu, Xiaohui Liang, Xueyi Dong, Nan Zhao

**Affiliations:** 1 Department of Pathology, Tianjin Medical University, Tianjin 300070, China; imcaozi@163.com (Z.C.); xiulanzhao@aliyun.com (X.Z.); wyft1022@163.com (Q.G.); liangxiaohui123@126.com (X.L.); dxy7235202@126.com (X.D.); zhaonantj@tmu.edu.cn (N.Z.); 2 Department of Pathology, General Hospital of Tianjin Medical University, Tianjin 300052, China; 3 Department of Pathology, Cancer Hospital of Tianjin Medical University, Tianjin 300060, China; Yanhuizhang015@163.com

The author wishes to make the following corrections to this paper [1]. The reasons for the corrections are: (1) error in representing Negative expression of Runx2 immunohistochemical staining picture in the old version of Figure 1, the mistake was due to mixing up the Negative expression of Runx2 immunohistochemical staining picture with the Negative expression of VE-cadherin immunohistochemical staining picture in the old version of Figure 1, it should be replaced with the correct Negative expression of Runx2 immunohistochemical staining pictures in the new version of Figure 1; (2) error in representing DAPI-stained SMMC7721-shRunx2-LGALS3 cell nuclei picture in the old version of Figure 6, the mistake was due to mixing up DAPI-stained SMMC7721-shRunx2-LGALS3 cell nuclei picture with DAPI-stained SMMC7721 cell nuclei picture, it should be replaced with the correct DAPI-stained SMMC7721-shRunx2-LGALS3 cell nuclei picture in the new version of Figure 6.

The correction does not change the conclusions of this manuscript. The authors would like to apologize for any inconvenience caused to the readers by these changes.

## Figures and Tables

**Figure 1:**
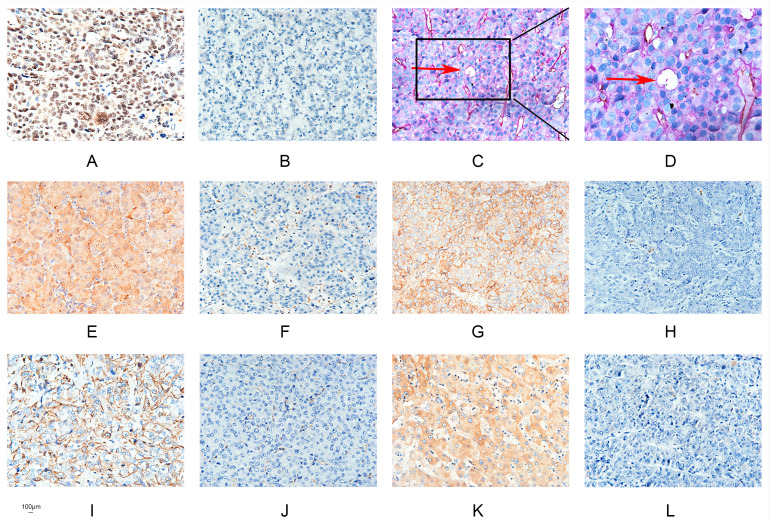
Hepatocellular carcinoma specimens were analyzed by immunohistochemistry. (**A**) Runx2 was predominantly localized in the nuclear of cancer cells (×200; bars 100 µm); (**B**) Negative expression of Runx2 (×200; bars 100 µm); (**C**) CD31/PAS double staining displayed VM channels (Red arrow) in Hepatocellular carcinoma specimens (×200; bars 100 µm); (**D**) VM channels (Red arrow)(×400; bars 100 µm); (**E**) Nuclear and cytoplasmic staining of Galectin-3 (×200; bars 100 µm); (**F**) Negative expression of Galectin-3 (×200; bars 100 µm); (**G**) Positive E-cadherin expression (×200; bars 100 µm); (**H**) Negative E-cadherin expression (×200; bars 100 µm); (**I**) Positive Vimentin expression (×200; bars 100 µm); (**J**) Negative Vimentin expression (×200; bars 100 µm); (**K**) Positive VE-cadherin expression (×200; bars 100 µm); (**L**) Negative VE-cadherin expression (×200; bars 100 µm).

**Figure 6:**
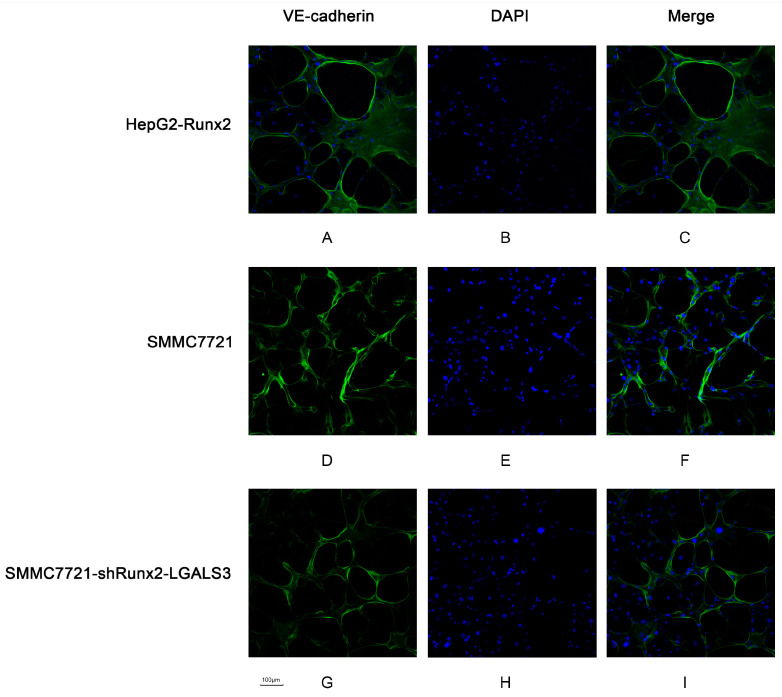
The VM-like tubes formed by HepG2-Runx2 cells, SMMC7721 cells and SMMC7721-shRunx2-LGALS3 cells were assessed by VE-cadherin immunofluorescence and confocal microscopy (×200). (**A**,**D**,**G**) VE-cadherin staining of the VM channel was concentrated in the wall of the tubes; (**B**,**E**,**H**) The cell nuclei were stained by DAPI; (**C**,**F**,**I**) The merged images showed that the expression of VE-cadherin in VM networks.
